# Stress and its association with involvement in online classes: a cross-sectional study among undergraduate students of a medical college in South India

**DOI:** 10.12688/f1000research.110920.1

**Published:** 2022-06-08

**Authors:** Rohith Motappa, Malavika Sachith, Pracheth Raghuveer

**Affiliations:** 1 Department of Community Medicine, Kasturba Medical College, Mangalore, Manipal Academy of Higher Education, Manipal, India; 2 Department of Community Medicine, Yenepoya Medical College, Yenepoya (Deemed to be University), Mangalore, India, Mangalore, Karnataka, 575003, India

**Keywords:** Online classes, Perceived stress, COVID 19

## Abstract

**Background: **With the implementation of lockdown and all students restricted to their houses, medical education has shifted towards the online mode. The objective of this study was to assess stress during the pandemic and the association between stress and involvement in online classes among students of a medical college in Mangalore, Karnataka, South India.

**Methods: **A cross-sectional study was conducted among 324 undergraduate students at a medical college in Mangalore, Karnataka, South India. The extent of stress was assessed using a perceived stress scale (PSS), and a questionnaire was used to identify different stressors and to understand the participant’s involvement in online classes.

**Results: **In this study, the mean perceived stress score was 21.66 ± 4. Moderate stress was observed in 262 (85%) students. The main stressors noted were inability to focus (173 (56.4%)) and fear of exams (153 (49.8%)). A significant association was noted between stress and involvement in online classes.

**Conclusions: **This study thereby highlights the need for more attention to the various stressors among students and for making online classes student friendly.

## Introduction

Coronavirus disease 2019 (COVID-19) was first reported in Wuhan, Hubei Province, China. On January 27 ^th^, 2020, the first case in India was reported in the state of Kerala. Thereafter, on March 11 ^th^, 2020, the World Health Organisation declared it a pandemic.

COVID-19 is a novel organism that spreads rapidly among people. Countries feared the doubling rate of the disease and the number of deaths due to it. Although people were being educated to practise preventive measures such as social distancing, wearing masks and sanitising hands, in countries such as India, the rates were still climbing. Hence, to break the chain of this infection, a nationwide complete lockdown was imposed in India on March 24 ^th^ 2020.

Lockdown is an emergency protocol that prevents people from leaving a given area. In the case of complete lockdown, only essential supplies such as grocery stores, pharmacies and banks continued to serve people.

India is the country with the largest proportion of the population at a young age. Hence, when lockdown was imposed, they were the worst hit, as this was a generation that has never sat idle but was always on the go. Additionally, the last pandemic faced by India was the 2009 Swine flu, ^1^ so this subject population is totally new to this disease and its preventive measures. As part of the lockdown, all educational institutions, including medical colleges, were shut down, and students were sent back home. Students are most affected by this, as they are losing out on their valuable school and college days. They are confined to the four walls of their households and are exposed to the outer world only virtually via the internet.

It was found that psychological symptoms such as depression and anxiety were elevated during the period of lockdown and isolation. ^2^ Student suicide was noted during this period, with the first case occurring in Kerala being a 15-year-old who committed suicide. ^3^

In studies conducted during the pandemic, it was found that stress, anxiety and depression due to COVID-19 were prevalent among the general population. ^4^ Among students, medical students were generally found to have higher stress than nonmedical students. ^5^ With the implementation of the lockdown, all the students were sent home, and online teaching was the only way to continue education.

Online education is an electronic learning tool that relies on the internet for teacher/student interaction and the distribution of class material. In this way, a student can turn anywhere with internet access and electricity into a virtual classroom. Although this method was introduced as an add on to traditional teaching and learning experience, with COVID-19 still prevalent throughout the world, it has become the new normal for generation Z.

Medical students are the upcoming doctors. Being at home and with an indefinite closure of colleges, there is a prevailing fear of the future among students. For the past 7–8 months, they were attending online classes. This has affected medical education with no more practical classes. This study aimed to assess stress during the pandemic and the association between stress and involvement in online classes among students of a medical college in Mangalore, Karnataka, South India.

## Methods

Institution Ethics Committee approval **(Yenepoya Ethics Committee-2, Protocol number: YEC2/622, dated 26/12/2020)** and permission from the institutional head were obtained before conducting the study. This is a cross-sectional study conducted among medical students studying Bachelor of Medicine and Bachelor of Surgery (MBBS) at Yenepoya Medical College situated in Mangalore taluk, Dakshina Kannada district, Karnataka, South India. The study was conducted from November to December 2020. Complete enumeration was used to enrol participants in this study, including participants studying in the first to final year in the medical college and the total sample size was 600. Approximately six attempts were made to reach out the participants through their online classes and WhatsApp groups, but we did not receive the desired response from the students. Thereby, the final sample size was accounted to 324.

A predesigned, validated, semi-structured questionnaire was used to capture information. ^6^ Demographic variables such as age, sex, religion, year of study and current place of stay were included. The Perceived Stress Scale (PSS), a classic instrument to assess stress, was also incorporated into the questionnaire. ^7^ The questions here ask about the feelings and thoughts of the participants during the last month. It is a 10-item questionnaire with responses in the form of how often participants did experience certain situations from 0-never to 4-very often. Scores ranging from 0 to 13 were considered low stress. Scores ranging from 14 to 26 were considered moderate stress. Scores ranging from 27 to 40 were considered high perceived stress. Questions pertaining to the participants’ involvement in online classes conducted by the college were also included. The Google Form also included a participant information sheet and informed consent. ^8^ All participants were voluntary, and it was described that the identity and the information given would remain strictly confidential. The Google Form responses were downloaded in a coma separated value (CSV) format, and the data captured were then cleaned. Data collected were analysed using SPSS Version 23. Descriptive statistics such as the mean, frequency and proportion were applied. The chi-square test was used to study the association between stress and involvement in online classes.

## Results

A total of 324 students responded to the survey, and 17 (5%) students did not give consent. The mean age of the students was 21 ± 2 years. The characteristics of the students are depicted in Table 1.

**Table 1.** Demographic characteristics of the participants (n = 307).

Variable	Frequency (n)	Percentage (%)
**Gender**		
Male	122	39.7
Female	185	60.3
**Religion**		
Hindu	113	36.8
Muslim	166	54.1
Christian	28	9.1
**Year of study**		
1 ^st^ year	128	41.7
2 ^nd^ year	117	38.1
3 ^rd^ year	34	11.1
4 ^th^ year	28	9.1
**Place of residence**		
Hostel	253	82.4
Day scholar	54	17.6

**Figure 1.**
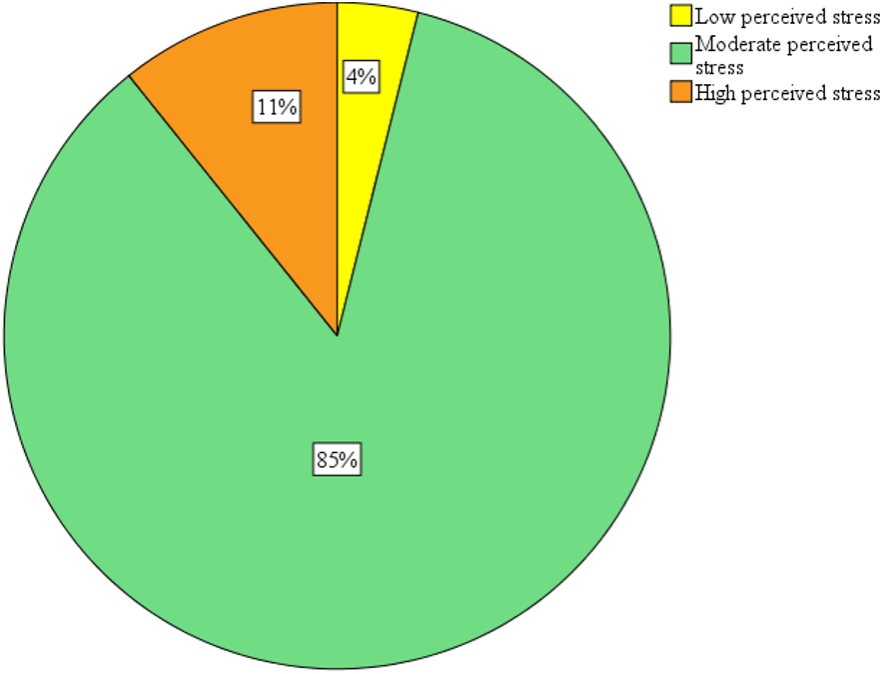
Stress level among medical students.

The mean perceived stress score was 21.66 ± 4. Out of 307 students, moderate stress was observed in 262 (85%) students ( Figure 1).

Among the various stressors identified, inability to focus and fear of exams were the most common stressors (173 (56.4%) and 153 (49.8%), respectively) ( Table 2), and other stressors were record completions, phone addiction, etc.

**Table 2.** Different stressors of students (n=307).

Variable *	Frequency (n)	Percentage (%)
1. Prolonged stay at home	98	31.9
2. Inability to attend classes	70	22.8
3. Difficulty in grasping	135	44
4. Inability to focus in studies	173	56.4
5. Fear of exams	153	49.8
6. Fear of COVID 19 infection	63	20.5
7. In adequate information of COVID-19	17	5.5
8. Financial situation of family	62	20.2
9. College fees	83	27

* Multiple response questions.

On assessing the preference for the mode of teaching among medical students pre- and post-pandemic, it was observed that the offline mode of teaching was preferred during both periods (226 (73.6%) and 170 (55.4%), respectively).

The association between perceived stress score and online classes is depicted in Table 3. There was a statistically significant association between perceived stress score and remembering content of online classes (p < 0.05), between perceived stress score and hours of classes attended (p < 0.05), and between perceived stress score and difficulty in concentrating online classes (p < 0.05).

**Table 3.** Association of perceived stress categories with involvement in online classes (n = 307).

	Mild stress	Moderate stress	High stress	Total	p value
**Do you remember the contents of online classes appropriately?**					
Yes	8	67	3	78	0.01
No	4	195	30	229	
**How many hours of online classes do you attend daily?**					
Less than 3 hours	8	159	12	179	0.02
More than 3 hours	4	103	21	128	
**Are you finding it difficult to concentrate in online classes?**					
Yes	6	198	30	234	0.01
No	6	64	3	73	
**Are you facing difficulty in understanding online classes?**					
Yes	4	164	26	194	0.08
No	8	98	7	113	
**Are you facing technological difficulty?**					
Yes	4	129	20	153	0.5
No	8	133	13	154	
**Are the hours of studying increased or decreased?**					
Increased	5	73	7	85	0.6
Same	2	42	8	52	
Decreased	5	147	18	170	

## Discussion

This study explored stress among 307 medical undergraduates of a medical college in South India and its association with involvement in online classes.

Although there are many studies assessing the stress level among medical students, this is one of the few studies conducted in South India aimed at identifying different stressors and their association with involvement in online classes during this COVID pandemic.

In this study, the mean perceived stress score was 21.66 ± 4, with moderate stress in 262 (85%) students. These findings were contrary to a study conducted by Joseph *et al* in which the mean stress score was 13.3 ± 4.2. ^9^ This could be attributed to the fact that our study was conducted during a period of pandemic with the whole world in immense stress.

The newer stressors due to online learning found in our study were inability to focus on studies, fear of exams, prolonged stay at home, etc., while previous studies suggest that potential stressors for medical students are academic workload and poor time management. ^10^^,^^11^ These findings were similar to those of a study conducted by Abdulghani *et al.* ^12^ COVID-related stressors were also noted, such as fear of infection and inadequate information on the disease, and these were consistent with a study conducted by Luberto *et al.* ^13^

Out of the 307 students who participated, 170 (55%) students experienced a decrease in overall time spent in studies after the implementation of online classes, which was consistent with the findings of previous studies. ^14^

The association between perceived stress score and remembering contents of online classes was found to be statistically significant in our study (p < 0.05). Among the 78 students who did not remember the contents of online classes, moderate stress was noted among 67 (85%) students. ^15^

In the present study, it was found that before the COVID pandemic and lockdown, only 55 (17.9%) students preferred the blended mode of classes with both online and offline modes. After the implementation of the lockdown, the number increased to 109 (35.5%). This is similar to a study conducted by Suryawanshi DM *et al.* ^16^

The study results highlight the fact that issues related to online teaching have become a new stressor for already stressed medical students. As online medical education is a new and evolving concept, the education system should make specific modules to help students. Additionally, students should be properly oriented for these classes, and individual-level monitoring must be done not just by teachers but also by parents. Tailor-made coping strategies must be developed for students to ease their stress. In the long run, colleges should modify the learning experience for each student with access to current technologies and the availability of resources for effective learning.

The strength of this study was that perceived stress scale, a standardised stress scale, was used in this study to access the stress level among the medical students and open-ended questions were asked to identify the different stressors. This study is reported according to the STROBE statement for reporting cross-sectional studies.

The study had several limitations. The study cannot be completely generalised, as it was conducted in a single private medical college. Here, most of the students were financially secure; hence, the study did not gather information about the needs of students with disabilities during the transition to online courses. As the study was based on a self-administered questionnaire, the chance of reporting bias could not be eliminated. Since the PSS questions are based on the experience of the students in the previous month, there is also a chance for memory or recall bias.

Further research is advocated for on stress among medical students and its association with online classes. In the “new normal” arena, research should also be directed toward teaching, learning, and evaluation strategies that can maximise learning results while minimising anxiety and negative psychological effects among students.

## Conclusion

Moderate perceived stress was observed in approximately 85.3% of students. Inability to focus on studies, fear of exams, and difficulty grasping are the key stressors. Due to this long-term lockdown as a result of the COVID-19 pandemic, the online mode of learning was the only way to continue medical education, which may cause further worsening of the psychological and learning behaviours of these students. Despite these difficulties, students’ faith in the efficiency of online medical education has grown as a result of their experiences during the first few weeks of the pandemic. While pandemics have historically posed difficulties, recognising these difficulties is the first step toward turning them into possibilities.

## Data availability

### Underlying data

Figshare: Stress and its association with involvement in online classes: a cross-sectional study among undergraduate students of a medical college in South India. https://doi.org/10.6084/m9.figshare.19375205.v1. ^17^

This project contains the following underlying data:

• Appraisal of stress V2.xlsx (This is the study participants data in the form of an excel sheet)

### Extended data

Figshare: Stress and its association with involvement in online classes: a cross-sectional study among undergraduate students of a medical college in South India-Questionnaire. https://doi.org/10.6084/m9.figshare.19431500.v1. ^6^

This project contains the following extended data:

• Questionnaire.docx (This is the questionnaire in word format)

Figshare: Consent form.docx. https://doi.org/10.6084/m9.figshare.19524058. ^18^

This project contains the following extended data:

• Consent form.docx (Blank copy of the consent form)

Data are available under the terms of the Creative Commons Attribution 4.0 International license (CC-BY 4.0).

## References

[1] Choudhry A Singh S Khare S : Emergence of pandemic 2009 influenza A H1N1, India. *Indian J. Med. Res.* 2012 Apr [cited 2021 May 10];135(4):534–537. 22664503 PMC3385239

[2] Brooks SK Webster RK Smith LE : The psychological impact of quarantine and how to reduce it: rapid review of the evidence. *Lancet.* 2020 [cited 2021 May 10];395:912–920. Lancet Publishing Group. 10.1016/S0140-6736(20)30460-8 32112714 PMC7158942

[3] Lathabhavan R Griffiths M : First case of student suicide in India due to the COVID-19 education crisis: A brief report and preventive measures. *Asian J. Psychiatr.* 2020 [cited 2021 May 10];53:102202. Elsevier B.V. 10.1016/j.ajp.2020.102202 32574939 PMC7297156

[4] Salari N Hosseinian-Far A Jalali R : Prevalence of stress, anxiety, depression among the general population during the COVID-19 pandemic: A systematic review and meta-analysis. *Global. Health. BioMed Central.* 2020 [cited 2021 May 10];16:1–11. 10.1186/s12992-020-00589-w 32631403 PMC7338126

[5] Aamir IS : Stress Level Comparison of Medical and Nonmedical Students: A Cross Sectional Study done at Various Professional Colleges in Karachi, Pakistan. *Acta Psychopathol.* 2017 Mar 31 [cited 2021 May 10];03(02). 10.4172/2469-6676.100080 Reference Source

[6] Motappa R Raghuveer P : Stress and its association with involvement in online classes: a cross-sectional study among undergraduate students of a medical college in South India-Questionnaire. figshare. 2022. 10.6084/m9.figshare.19431500.v1 PMC9263575 35860480

[7] State of New Hampshire Employee Assistance Program: Perceived Stress Scale Score Cut Off. *State New Hampsh Empl Assist Progr.* 1983;2.

[8] Motappa R Raghuveer P : Stress and its association with involvement in online classes: a cross-sectional study among undergraduate students of a medical college in South India- Consent form.docx. figshare. 2022. 10.6084/m9.figshare.19524058.v1 PMC9263575 35860480

[9] Joseph N Joseph N Panicker V : Assessment and determinants of emotional intelligence and perceived stress among students of a medical college in south India. *Indian J. Public Health.* 2015;59(4):310–313. 10.4103/0019-557X.169666 26584173

[10] Chowdhury R Mukherjee A Mitra K : Perceived psychological stress among undergraduate medical students: Role of academic factors. *Indian J. Public Health.* 2017;61(1):55–57. 10.4103/0019-557X.200253 28218165

[11] Hill MR Goicochea S Merlo LJ : In their own words: stressors facing medical students in the millennial generation. *Med. Educ. Online.* 2018;23(1). 10.1080/10872981.2018.1530558 30286698 PMC6179084

[12] Abdulghani HM Sattar K Ahmad T : Association of covid-19 pandemic with undergraduate medical students’ perceived stress and coping [response to letter]. *Psychol. Res. Behav. Manag.* 2020;13:1101–1102. 10.2147/PRBM.S292018 33273871 PMC7708311

[13] Luberto CM Goodman JH Halvorson B : Stress and Coping Among Health Professions Students During COVID-19: A Perspective on the Benefits of Mindfulness. *Glob. Adv. Heal. Med.* 2020;9:2164956120977827. 10.1177/2164956120977827 33403158 PMC7739073

[14] Kapasia N Paul P Roy A : Impact of lockdown on learning status of undergraduate and postgraduate students during COVID-19 pandemic in West Bengal, India. *Child Youth Serv. Rev.* 2020;116(June):105194. 10.1016/j.childyouth.2020.105194 32834270 PMC7308748

[15] Meo SA Abukhalaf AA Alomar AA : COVID-19 Pandemic: Impact of Quarantine on Medical Students’ Mental Wellbeing and Learning Behaviors. *Pak. J. Med. Sci.* 2020 [cited 2020 Oct 7];36:S43–S48. 10.12669/pjms.36.COVID19-S4.2809 32582313 PMC7306952

[16] Suryawanshi DM Venugopal R : Preferences, perceptions and barriers to E-learning among medical students during COVID-19 pandemic lockdown in India. *Int. J. Community Med. Public Heal.* 2020;7(10):4100. 10.18203/2394-6040.ijcmph20204383

[17] Raghuveer P Motappa R : Stress and its association with involvement in online classes: a cross-sectional study among undergraduate students of a medical college in South India. figshare. [Dataset]. 2022. 10.6084/m9.figshare.19375205.v1 PMC9263575 35860480

