# Supplementary material for: Stress and its association with involvement in online classes: a cross-sectional study among undergraduate students of a medical college in South India
Source: F1000Res. 2022 Jun 8;11:630. [Version 1] doi: 10.12688/f1000research.110920.1 (PMC9263575; doi:10.12688/f1000research.110920.1)
Supplement: Peer review history

# Peer review: Reviewer response for version 1

**DOI:** 10.5256/f1000research.122579.r141242

**Published:** 2022-07-07

**Authors:** Padmini Thalanjeri

**Affiliations:** 1 Department of Physiology, Yenepoya Medical College, Yenepoya (Deemed to be University), Mangalore, Karnataka, India

This study is a cross sectional study done on undergraduate medical students to determine the academic stress due to online curricular transactions during COVID times. It has shown that the online mode of learning created a significant increase in stress scores thus highlighting the need for more studies on various stressors and to pave the way forward for making online learning more student friendly.

 Minor comments:

1. In the results section of the abstract, please add more findings relevant to the study objectives.
2. The introduction may be further strengthened by adding studies carried out on stress among medical students particularly during the COVID pandemic.
3. Kindly modify the conclusion and make it specific to the study objectives.
4. What are the recommendations based on the study findings?

Is the work clearly and accurately presented and does it cite the current literature?

Yes

If applicable, is the statistical analysis and its interpretation appropriate?

Yes

Are all the source data underlying the results available to ensure full reproducibility?

Yes

Is the study design appropriate and is the work technically sound?

Yes

Are the conclusions drawn adequately supported by the results?

Yes

Are sufficient details of methods and analysis provided to allow replication by others?

Yes

Reviewer Expertise:

Medical education, Bioethics

I confirm that I have read this submission and believe that I have an appropriate level of expertise to confirm that it is of an acceptable scientific standard.

# Peer review: Reviewer response for version 1

**DOI:** 10.5256/f1000research.122579.r140148

**Published:** 2022-06-20

**Authors:** Chonnakarn Jatchavala

**Affiliations:** 1 Department of Psychiatry, Prince of Songkla University, Songkhla, Thailand

Introduction:

 -Paragraph 1, not necessary to mention Wuhan, when 1st detected in India and your area.

 -Before indicating online education, please briefly explain your curriculum of medicine (MBBS?) for years 1,2,3, and 4. What about studying pre-clinic and clinical practice study?

 -Speaking about "online education or digital psychiatry", please reference and describe "For example, implementing digital training programs may significantly improve the level of knowledge in psychiatry in Asia-pacific region"

 Please ref: https://onlinelibrary.wiley.com/doi/full/10.1111/appy.12501

 -How long did the lockdown your medical students experienced that year last?

 -Do you have any rate of Covid-19 infection in medical students/ university students in the area?

 Methods:

 -Please identify the validity of the tools.

 Results:

 -It is quite short, with many results you can describe interestingly. Data in Table 3 is confusing, you should explain every item's result and discuss it.

 Discussion:

 -What is your suggestion for your medical students from your paper and other research? It will be very good if you can sum it up in a box.

 Please reference: https://ps.psychiatryonline.org/doi/abs/10.1176/appi.ps.202000774

 https://link.springer.com/article/10.1007/s40596-021-01482-3

Is the work clearly and accurately presented and does it cite the current literature?

Partly

If applicable, is the statistical analysis and its interpretation appropriate?

Yes

Are all the source data underlying the results available to ensure full reproducibility?

Partly

Is the study design appropriate and is the work technically sound?

Yes

Are the conclusions drawn adequately supported by the results?

Yes

Are sufficient details of methods and analysis provided to allow replication by others?

Partly

Reviewer Expertise:

Psychiatry, mental health, suicide, students

I confirm that I have read this submission and believe that I have an appropriate level of expertise to confirm that it is of an acceptable scientific standard.

## References

[1] : Training and education in digital psychiatry: A perspective from Asia-Pacific region. *Asia Pac Psychiatry* .2021;13(4) : 10.1111/appy.12501 e12501 10.1111/appy.12501 34873845PMC9285069

[2] : Challenges and Opportunities of Psychiatric Training During COVID-19: Early Career Psychiatrists’ Perspective Across the World. *Academic Psychiatry* .2021;45(5) : 10.1007/s40596-021-01482-3 656-657 10.1007/s40596-021-01482-3 PMC814758334032993

[3] : Scaling Up Global Mental Health Services During the COVID-19 Pandemic and Beyond. *Psychiatric Services* .2022;73(2) : 10.1176/appi.ps.202000774 231-234 10.1176/appi.ps.202000774 34235945

# Response

**Published:** 2022-06-24

**Authors:** Pracheth Raghuveer

Thank you very much for the detailed review. Please find our response to your comments.

 Introduction:

**-Paragraph 1, not necessary to mention Wuhan, when 1st detected in India and your area.**

 Response: It will be modified as commented. 

**-Before indicating online education, please briefly explain your curriculum of medicine (MBBS?) for years 1,2,3, and 4. What about studying pre-clinic and clinical practice study?**

 Response: In India, the MBBS curriculum is structured in this way. In the first professional year, pre-clinical subjects like Anatomy, Physiology and Biochemistry are the core subjects. In the second professional year,  para-clinical subjects like Pathology, Microbiology, Pharmacology are the core subjects and in the third professional year, Community Medicine, ENT, Ophthalmology and Forensic  Medicine are the core-subjects. In the final year, clinical subjects like Medicine, Surgery, OBG and Pediatrics are the core subjects. The teaching involves an integrated approach with emphasis on small group teaching and early clinical exposure.  This is as per the latest Competency Based Medical Education mandated by the National Medical Commission of India in the year 2019.

**-Speaking about "online education or digital psychiatry", please reference and describe "For example, implementing digital training programs may significantly improve the level of knowledge in psychiatry in Asia-pacific region"**

**Please ref: https://onlinelibrary.wiley.com/doi/full/10.1111/appy.12501**

 Response: Thanks. This suggestion will be incorporated. 

**-How long did the lockdown your medical students experienced that year last?**

 Response:  In India, the nation-wide lockdown lasted from 25 March 2020 to 31 May 2020. This was followed by a gradual ease of restrictions. 

**-Do you have any rate of Covid-19 infection in medical students/ university students in the area?**

 Response: Yes, we do have the data of COVID-19 infection among the medical students in our area. The data may be available on request from the district health officials. However, this study  was carried out during the time when the students were off-campus, that is, the lockdown period of 25 March-31 May, 2020. 

**Methods:**

**-Please identify the validity of the tools.**

 Response: A pre-tested, pre-designed and validated proforma was used to collect the data. 

**Results:**

**-It is quite short, with many results you can describe interestingly. Data in Table 3 is confusing, you should explain every item's result and discuss it.**

 Response: Thanks for this comment. Table 3 denotes the association of perceived stress categories with the involvement of online classes. Detailed description will be given in the subsequent version. 

**Discussion:**

**-What is your suggestion for your medical students from your paper and other research? It will be very good if you can sum it up in a box.**

**Please reference: https://ps.psychiatryonline.org/doi/abs/10.1176/appi.ps.202000774**

**https://link.springer.com/article/10.1007/s40596-021-01482-3**

 Response: Thanks. This will be summed up in a box as suggested.
